# Anti-addiction Drug Ibogaine Prolongs the Action Potential in Human Induced Pluripotent Stem Cell-Derived Cardiomyocytes

**DOI:** 10.1007/s12012-016-9366-y

**Published:** 2016-03-28

**Authors:** Lena Rubi, Daniel Eckert, Stefan Boehm, Karlheinz Hilber, Xaver Koenig

**Affiliations:** Department of Neurophysiology and – Pharmacology, Center for Physiology and Pharmacology, Medical University of Vienna, Schwarzspanierstrasse 17, 1090 Vienna, Austria

**Keywords:** Action potential repolarization, Anti-addiction drug ibogaine, Cardiac arrhythmias, Drug-induced QT interval prolongation, Human cardiomyocytes, Noribogaine

## Abstract

Ibogaine is a plant alkaloid used as anti-addiction drug in dozens of alternative medicine clinics worldwide. Recently, alarming reports of life-threatening cardiac arrhythmias and cases of sudden death associated with the ingestion of ibogaine have accumulated. Using whole-cell patch clamp recordings, we assessed the effects of ibogaine and its main metabolite noribogaine on action potentials in human ventricular-like cardiomyocytes derived from induced pluripotent stem cells. Therapeutic concentrations of ibogaine and its long-lived active metabolite noribogaine significantly retarded action potential repolarization in human cardiomyocytes. These findings represent the first experimental proof that ibogaine application entails a cardiac arrhythmia risk for humans. In addition, they explain the clinically observed delayed incidence of cardiac adverse events several days after ibogaine intake. We conclude that therapeutic concentrations of ibogaine retard action potential repolarization in the human heart. This may give rise to a prolongation of the QT interval in the electrocardiogram and cardiac arrhythmias.

## Introduction

The plant alkaloid ibogaine exerts convincing anti-addictive properties, but has never been approved as anti-addiction medication [1, 2]. Largely because of ibogaine’s status as banned substance in the USA since 1970, the further development of the alkaloid’s use in addiction therapy took place outside conventional clinical and medical settings [1]. Ten years ago, Frank Vocci, at that time director of anti-addiction drug development at the National Institute on Drug Abuse, termed ibogaine therapy “a vast, uncontrolled experiment” [3]. Nevertheless, the ibogaine “medical subculture” has continued to grow, with dozens of alternative medicine clinics operating worldwide [1, 2].

Recently, alarming reports of life-threatening cardiac arrhythmias and sudden death cases [4–12], temporally associated with the ingestion of ibogaine, have accumulated. We [13–16] and others [17] hypothesized that these were related to the drug’s propensity to block human ether-a-go-go-related gene (hERG) potassium channels in the heart, which can result in retardation of ventricular action potential (AP) repolarization and prolongation of the QT interval in the electrocardiogram (ECG) [18]. QT prolongation—indeed observed in people after ibogaine intake (e.g. [2, 4, 5])—is known to be associated with an increased risk of life-threatening torsade de pointes (TdP) arrhythmias [18].

Up to now, the inhibition of currents through heterologously expressed hERG channels by ibogaine (IC_50_ value, drug concentration needed for half-maximum inhibition = 3–4 µM [13, 14, 17]) remains the sole experimental evidence for the drug’s proarrhythmic potential in humans; data on human cardiomyocytes are still lacking. Moreover, two observations have cast reasonable doubts on ibogaine’s cardiotoxic effects: first, although hERG channel inhibition should retard AP repolarization, the drug significantly shortens AP duration in guinea pig ventricular cardiomyocytes [14]), a well-established model system for human myocytes. Secondly, cardiac adverse events in humans often occur many hours or even several days after ibogaine intake [2], albeit the drug has a half-life in human plasma of 4–7 h only [2, 19].

## Methods

Details of our experimental procedures are described elsewhere [14]. Action potentials (APs) were recorded from “ventricular-like” human induced pluripotent stem cell-derived cardiomyocytes (hiPS-CM, Cellectis, Sweden) in the current-clamp mode of the whole-cell patch clamp technique at room temperature (22 ± 1 °C). Cardiomyocytes were classified as ventricular-like if their APs showed a distinct “shoulder” (plateau or flat repolarization phase) prior to a final steep phase of repolarization. APs were elicited at 1 Hz by rectangular current pulses of 4 ms duration at 125 % threshold level. The pipette solution contained 10 mM NaCl, 140 mM KCl, 2 mM EGTA, 1 mM MgCl_2_, 0.1 mM Na-GTP, 5 mM Mg-ATP, 10 mM Hepes, and pH = 7.2 adjusted with KOH. The cells were bathed in 140 mM NaCl, 4 mM KCl, 2 mM CaCl_2_, 2 mM MgCl_2_, 5 mM HEPES, 5 mM glucose, and pH = 7.4 adjusted with NaOH.

Whole-cell currents through hERG potassium channels heterologously expressed in TSA-201 cells were recorded as in our previous studies [13, 14].

## Results and Discussion

**Fig. 1:**
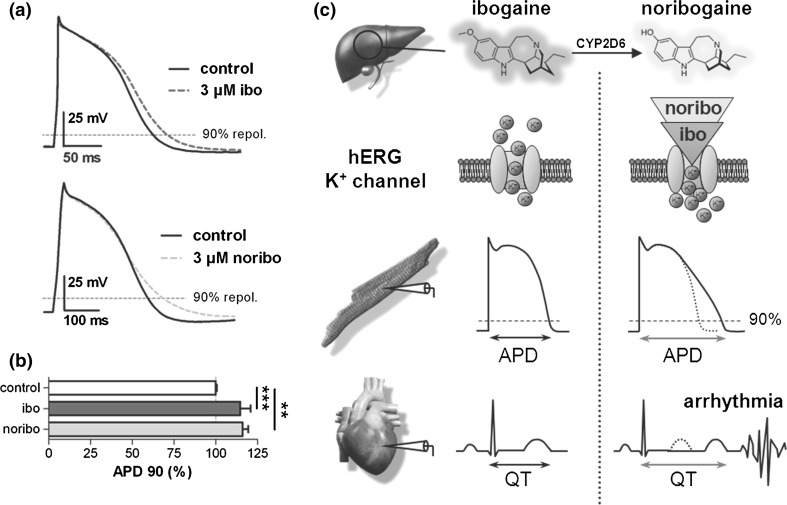
Ibogaine and noribogaine impair the electrophysiology of the human heart. **a** Action potential (AP) recordings from human cardiomyocytes under control conditions and after superfusion with bath solution containing 3 µM ibogaine (ibo, *top*) or noribogaine (noribo, *bottom*). **b** Analysis of the retardation of AP repolarization by ibogaine and noribogaine. APD90 (mean ± SD, *n* = 6–16), AP duration at 90 % repolarization. **(*p* < 0.01) and ***(*p* < 0.001), significantly different from control (paired Student’s *t* test performed on raw data prior to normalization). The absolute APD90 values (in ms) were 282 ± 147 for control and 322 ± 165 for 3 µM ibogaine, as well as 264 ± 137 for control and 305 ± 155 for 3 µM noribogaine. APD50, AP duration at 50 % repolarization, was neither altered by 3 µM ibogaine (*p* = 0.64) nor noribogaine (*p* = 0.39). **c** Proposed mechanism of cardiac arrhythmia generation after ibogaine intake. The cartoon summarizes the causal sequence of drug effects at the ion channel, cellular, and organ level: both ibogaine and noribogaine, the latter generated from ibogaine by CYP2D6 enzymes in the liver [21, 22], block hERG potassium channels and thereby retard the repolarization phase of the ventricular AP. As a consequence, the QT interval in the ECG gets prolonged, and finally, cardiac arrhythmias emerge

**Fig. 2:**
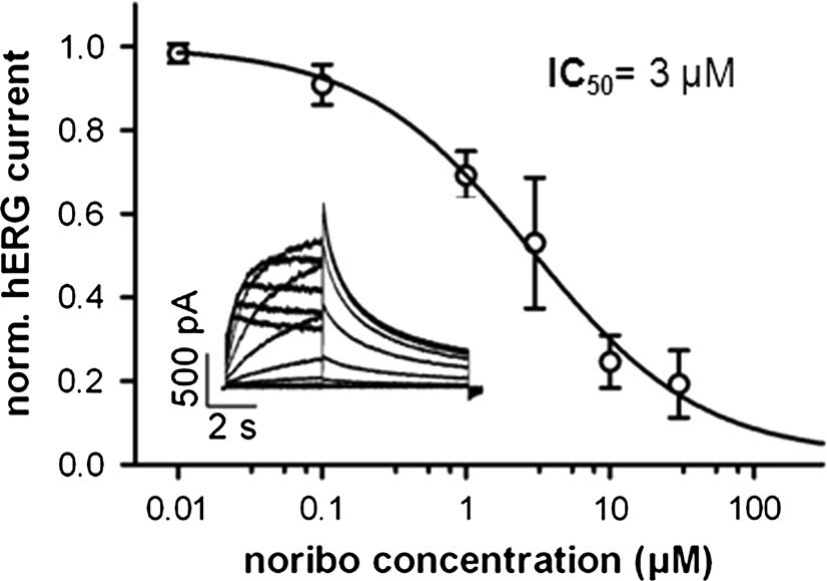
Concentration dependence of hERG current inhibition by noribogaine (noribo). hERG channels were heterologously expressed in TSA-201 cells, and hERG current examples are shown in the *inset*. IC_50_, drug concentration needed for half-maximum inhibition. Data points represent mean ± SD (*n* = 7–10)

By assessing the effects of ibogaine and its main metabolite noribogaine on APs in cardiomyocytes derived from human induced pluripotent stem cells, we provide the first experimental proof that ibogaine application does indeed entail a cardiac risk for humans, and we further unravel the mystery of the surprising longevity of the drug’s potential to cause arrhythmias. Thus, Fig. 1 shows that 3 µM ibogaine, a concentration at the IC_50_ value for hERG channel inhibition [13, 14, 17] and well within the free plasma concentration range reached in humans after drug intake (up to 11 µM) [14], significantly prolongs the AP and flattens its repolarization phase in human cardiomyocytes [Fig. 1a (top), b]. This effect was fully reversible after washout of the drug (data not shown). Similar to ibogaine, also its long-lived active metabolite noribogaine (plasma half-life, 28–49 h [20]) prolonged the human cardiac AP [Fig. 1a (bottom), b]. This observation goes along with noribogaine’s inhibitory action on hERG potassium channels (IC_50_ = 3 µM: Fig. 2; [17]), which very closely resembles hERG inhibition by ibogaine [13, 14, 17]. It further provides an explanation for the delayed incidence of cardiac adverse events, which may occur even several days after ibogaine intake.

Based on these unprecedented findings, we propose the following sequence to explain the cardiotoxicity associated with ibogaine ingestion (Fig. 1c): (1) inhibition of repolarizing hERG potassium channels by ibogaine and its metabolite noribogaine, followed by (2) retardation of ventricular AP repolarization, and finally (3) prolongation of the QT interval in the electrocardiogram (ECG), ultimately paving the way for life-threatening TdP arrhythmias. Because of its long half-life in human plasma [20], we consider noribogaine, rather than ibogaine itself, as the crucial molecule triggering this sequence of deleterious events.

Since drug-induced AP prolongation in ventricular cardiomyocytes directly correlates with prolonged QT intervals in the ECG [18], the actions of ibogaine and noribogaine reported herein can explain previously published case reports of QT interval prolongation triggered by single doses of ibogaine and lasting several days (e.g. [2, 4, 5]). hERG channel inhibition and concomitant retardation of AP repolarization caused by therapeutic plasma concentrations of any drug are well-known predictors of an increased risk for TdP arrhythmias and sudden cardiac death [18]. Moreover, formerly approved drugs with ibogaine-like effects on in vitro cardiac electrophysiology, e.g. cisapride and astemizole, have been withdrawn from the market because of the unbearable TdP arrhythmia risk associated with their application [18].

We therefore conclude that the use of ibogaine, at doses currently administered to drug addicts, must be considered a dangerous practice. Arrhythmias may emerge due to drug-induced QT interval prolongation resulting from retarded cardiac action potential repolarization.
